# Subcycle-resolved probe retardation in strong-field pumped dielectrics

**DOI:** 10.1038/ncomms8746

**Published:** 2015-07-13

**Authors:** Aseem Prakash Pati, Imam Setiawan Wahyutama, Adrian Nikolaus Pfeiffer

**Affiliations:** 1 Institute for Optics and Quantum Electronics, Abbe Center of Photonics, Friedrich Schiller University, Max-Wien-Platz 1, 07743 Jena, Germany

## Abstract

The response of a bulk dielectric to an intense few-cycle laser pulse is not solely determined by the pulse envelope, but also by ultrafast processes occuring during each optical cycle. Here, a method is presented for measuring the retardation of a probe pulse in a strong-field pumped, bulk dielectric with subcycle resolution in the pump–probe delay. Comparisons to model calculations show that the measurement is sensitive to the timing of the electronic Kerr response. When conduction band states are transiently populated at the crests of the laser field, the measurement is also sensitive to the interband dephasing time.

## Abstract (web summary)

At the ultrafast timescale the propagation of light pulses through a dielectric material is not only determined by the envelope, but also by nonlinear interactions that evolve within one optical cycle. Here, the authors demonstrate a method to determine the subcycle-resolved delay to a probe pulse in ultrafast, high-field pump–probe experiments.

## Graphical abstract

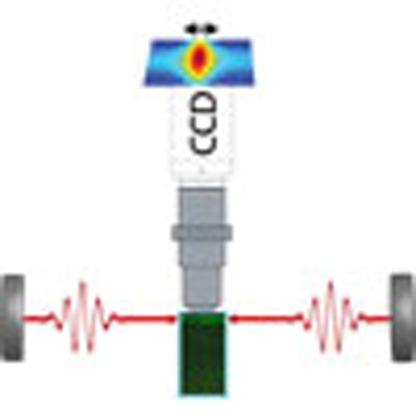

The investigation of electronic processes on the attosecond timescale has led to numerous remarkable results for atoms, molecules, nanostructures and surfaces^1^. Studies on bulk solids are being performed only since recently^2^,^3^,^4^,^5^,^6^,^7^,^8^,^9^,^10^,^11^,^12^,^13^,^14^,^15^,^16^,^17^, and the arising picture is extremely promising. It was shown that electric currents in a bulk dielectric could be created and manipulated within one optical cycle of a femtosecond laser pulse^5^,^6^,^7^. Laser pulses in the midinfrared^3^,^4^ and in the terahertz spectral range^15^,^18^ have been used for high-order harmonic generation (HHG) in bulk crystalline solids, potentially leading to next-generation schemes for attosecond pulse generation. Next to this technological potential, the ultrafast electronic response of dielectrics to strong-field pulses may advance our fundamental understanding of light–matter interaction. For example, electrons can reach the edges of the first Brillouin zone at these intensities and lead to dynamical Bloch oscillations in crystals^15^,^16^, an effect that was thought to be impossible to observe in bulk solids because of scattering.

The experimental progress is hampered by the fact that many established methods of attosecond physics, especially those utilizing the detection of photoelectrons, are not applicable to bulk samples. New experimental methods are needed both to advance the fundamental understanding of strong-field processes in solids and to develop new technological schemes to exploit them. To date it is an open question how the feasibility to manipulate electric currents within one optical cycle could ultimately be employed for ultrafast signal processing. Schemes for signal processing will need to be developed, and most likely the coherence of the underlying processes will be a critical quantity.

So far, little is known about the coherence properties of strong-field processes in solids. In a theoretical model, a transient conductivity is induced by transient population transfer from the valence band to the conduction band^16^, sometimes referred to as virtual excitation^19^. The coherence of these virtual excitations decays at a rate determined by the interband dephasing time. However, as mentioned in ref. 16, little is known about ultrafast dephasing in dielectrics. In ref. 13, a novel way to determine the dephasing time is suggested by comparison of theoretical analysis of HHG in solids to experiments, which leads to an estimate of about 4 fs for ZnO (ref. 13). Knowledge about the dephasing time is of great importance for HHG in solids, as it determines the contribution of high-order returns^13^.

Here, an experimental method is presented which delivers time-resolved information about strong-field processes that occur in dielectric solids during one laser cycle. The method is based on the well-known retardation of a probe pulse in the presence of a strong pump pulse^20^. A close-to-collinear alignment of pump and probe beams facilitates the detection of subcycle dynamics with respect to an absolute time reference given by the interference of the pump and the probe pulse. Comparisons to calculations show that the measurement is sensitive to the timing of the electronic Kerr response. To investigate the influence of strong-field effects, model calculations are performed based on band structures. This is based on the assumption that the observed effects do not depend on the crystallinity of the dielectric, as it was found and reasoned for the strong-field regime in the study on optical-field-induced currents in dielectrics^5^. Comparing the subcycle-resolved measurement of the probe retardation in borosilicate glass due to a 7-fs pulse at a peak intensity of 2.3 TW cm^−2^ to a calculation based on a two-band model reveals that the transient conduction band population affects the measurement in a characteristic way. Moreover, the measurement is sensitive to the interband dephasing time and hence delivers information about the coherence behaviour of the strong-field induced conductivity.

## Results

### Subcycle resolution in pump–probe experiments

It is known since a long time that a strong laser pulse can transiently modify the refractive index of a nonlinear medium and thereby influence the propagation of a probe pulse (‘weak-probe retardation'^20^). An induced phase shift in the probe pulse is often referred to as cross-phase modulation, an induced amplitude change is often referred to as two-beam coupling^21^. Under phase matching conditions, these effects are accompanied by third-harmonic generation and four wave mixing. All these effects can be explained via the third-order nonlinearity of the medium. Very recently, it was discovered that the mechanism of frequency transformation changes when the pump pulse intensity is sufficiently high for ionization of the solid: an optical signal was observed and assigned to free-electron density changes on the attosecond timescale^2^. In analogy to that effect, here it is presented how the process of cross-phase modulation changes when the pump pulse intensity is strong enough to create free electrons in virtual states.

**Figure 1:**
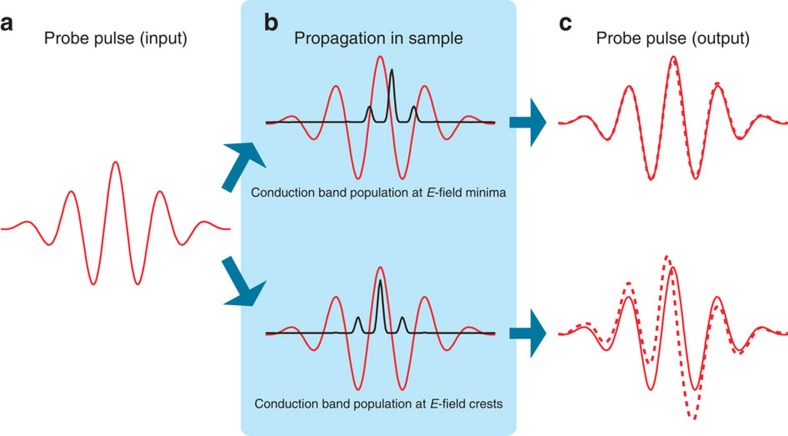
The principle of subcycle-resolved probe-retardation measurements. A femtosecond input pulse in the few-cycle regime (red line, **a**) propagates through a dielectric bulk sample (**b**). The conduction band population (black line) varies with a periodicity of half the optical period. The output pulse (red dashed line, **c**) is almost identical to the input pulse (red solid line, shown for reference) in case that the conduction band is populated at minima of the electric field strength. In case that the conduction band is populated at crests of the electric field strength, the output pulse is strongly modified. The physical quantities here are not to scale but for illustration only.

The principle how the transient electron band population affects the probe retardation is illustrated in Fig. 1. In a very simplified view, the dielectric sample exhibits a time-dependent density of free electrons, following the electric field strength of a strong pump pulse. If the free-electron density peaks at local minima of the probe field strength, then the probe pulse experiences negligible changes during propagation, because the probe field accelerates the electrons only very little. In contrast, if the free-electron density peaks at local maxima of the probe field strength, then the probe pulse experiences significant changes during propagation. Hence, the subcycle-resolved measurement of the probe retardation carries information about the transient electron density.

The subcycle-resolved probe-retardation measurement holds fundamental difficulties for an experimental implementation. In a non-collinear alignment of the pump and the probe beam, the wavefronts of the pulses intersect each other in the focus, such that subcycle-dependent phenomena are averaged out in the far field. In a collinear alignment, the pump and the probe beam need to be separated after the interaction through their wavelength or through their polarization. Separation through wavelength is not possible for broadband pulses with sufficient intensity for frequency transformation. Separation through polarization is usually accompanied by heavy dispersive pulse broadening for polarizers with acceptable extinction ratios, in addition to complications for the measurement of the absolute time reference described below. Here, a close-to-collinear alignment is used, where the crossing angle of pump and probe beams is small enough to maintain subcycle-dependent phenomena, but big enough to allow a spatial separation of pump and probe after their interaction.

### Experimental setup

**Figure 2:**
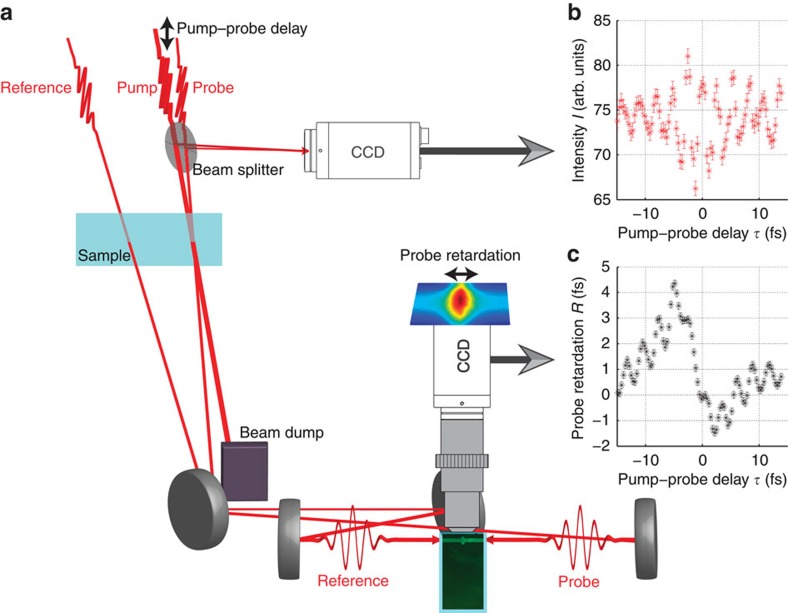
The experimental setup for subcycle-resolved probe-retardation measurements. (**a**) Femtosecond pump and probe pulses are focused into a bulk dielectric sample with a variable delay in a close-to-collinear alignment. The probe retardation is determined by imaging the fluorescence from a head-on collision of the probe pulse with a reference pulse inside a cuvette filled with Fluorescein. In front of the sample, a beamsplitter steers a fraction of the pump and probe pulses to a camera, which measures the intensity *I* (**b**) to give an absolute time reference for the subcycle-resolved probe-retardation measurement (**c**).

A 7-fs pulse with a peak wavelength at 820 nm is generated by a commercial femtosecond laser system and split into three collinearly polarized copies for pump, probe and reference pulses (Fig. 2). The pulses are focused into a bulk sample of borosilicate glass (Schott D 263 M, thickness 0.145 mm), where the pump pulse reaches a peak intensity of 2.3 TW cm^−2^, probe and reference pulses reach a peak intensity of 0.05 TW cm^−2^. Pump and probe pulses have a variable delay and overlap in the focus, and D-shaped mirrors are used to achieve a very small crossing angle of about 0.3° between them. No permanent modifications of the optical properties of the sample are observed, signalling that the pump intensity is well below the damage threshold through generation of an electron-hole plasma or an electron-ion plasma^22^. This is affirmed by the fact that the transmission of the pump beam alone without the presence of the probe pulse did not measurably depend on the pump intensity^22^, showing no significant nonlinear absorption. Simulations (see Methods section) show that the phase evolution of the pump pulse as it propagates through the sample does depend nonlinearly on the pump intensity, but this effect is smaller than the induced phase shift in the probe pulse, as it is expected in cross-phase modulation^20^.

Before the focus, a beamsplitter directs a small fraction of the pump and probe beams to a camera, placed at the focal distance. The camera records the intensity *I*, which is the sum of all the pixel values contained in a circle with 60 μm diameter centred on the focal spot of the probe beam. The intensity *I* depends on the pump–probe delay, because the beam crossing angle is small enough to not average out constructive interference (for pump–probe delays of integer multiples of the optical period time, leading to local maxima of *I*) and destructive interference (for pump–probe delays of odd multiples of the optical half-period time, leading to local minima of *I*). In this sense, the intensity *I* gives an absolute time reference between the pump and the probe pulse through interference. After the interaction in the bulk sample, the probe and the reference pulses are focused head-on into a custom cuvette containing a fluorescent medium (Fluorescein). The cuvette is designed for minimal dispersive pulse broadening (145 μm entrance window thickness and 145 μm inner spacing). The fluorescence is imaged with a microscope objective onto a camera. Through two-photon absorption, the temporal overlap of the probe and the reference pulses is mapped to the spatial domain: the spatial location of the maximum fluorescence shifts in space upon temporal retardation of the probe pulse. The probe retardation *R* is determined from the microscope image as described in the Methods section.

### Comparison to simulations

**Figure 3:**
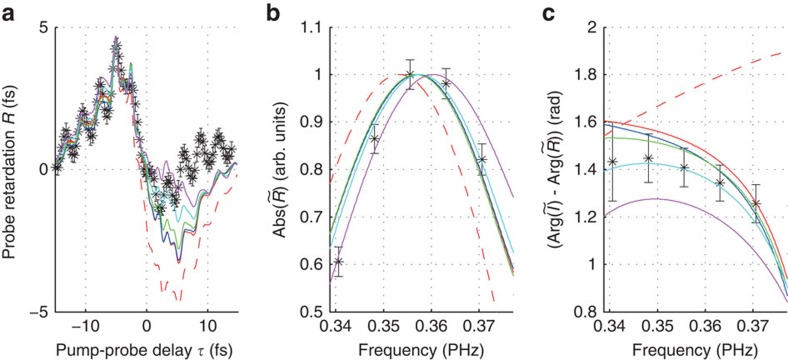
Subcycle-resolved probe-retardation measurement. The data (asterisks) is compared with the calculations (solid lines). The calculation without inclusion of transient conduction band population is marked by the red lines (red solid line for δ*t*=0 and red dashed line for δ*t*=100 as). The calculation with inclusion of transient conduction band population is marked by the blue solid line (*T*_2_=∞, *d*=0.15 a.u., δ*t*=0), the green solid line (*T*_2_=10 fs, *d*=0.15 a.u., δ*t*=0), the cyan solid line (*T*_2_=3.3 fs, *d*=0.15 a.u., δ*t*=0) and the magenta solid line (*T*_2_=2 fs, *d*=0.15 a.u., δ*t*=0). (**a**) The pump–probe delay scan of the probe retardation (for positive pump–probe delays the pump pulse is later in time), (**b**) the absolute of the Fourier transform and (**c**) the phase difference to the absolute time reference given by the intensity plotted in Fig 2b. For the calculation of the error bars, see Methods section.

To get insight into the mechanism that underlies the subcycle effects of the probe retardation, the propagation through the bulk sample is calculated numerically. In all calculations, the linear dispersion and the third-order nonlinearity *χ*^(3)^ of the material are included (see Methods section). This reproduces the main features of the measured probe retardation already reasonable well (Fig. 3). Oscillations are visible at a period that equals the optical period of the laser (about 2.7 fs). There are also oscillations at half the period of the optical cycle, but they are mostly averaged out due to the finite temporal resolution inherent to the pump-probe geometry.

The third-order nonlinearity *χ*^(3)^ consists of an electronic and a nuclear (that is, Raman) contribution. The electronic response is usually assumed to be instantaneous, and it was shown by femtosecond two-beam coupling that the response time is well below 1 fs, with no lower limit specified^23^. To investigate if finite response times of the electronic nonlinearity influence the subcycle-resolved probe retardation, calculations are performed where a delay δ*τ* is included in the third-order electronic response (see Methods section). A calculation with δ*τ*=100 as (the third-order electronic response follows the electric field of the laser pulse with a delay of 100 as) shows that the subcycle-resolved probe retardation is indeed very sensitive to the timing of the nonlinear response.

Due to the high peak intensity of the pump pulse, it is expected that the third-order nonlinearity fails to adequately describe the process. Nonlinear ionization is a strong-field process that influences the pulse propagation^24^. In addition, higher-order nonlinearities in the response of the bound electrons may have to be considered. For gases, a very controversial discussion started recently about the influence of higher-order Kerr coefficients versus the influence of the free-electron plasma created by ionization^21^,^25^,^26^. Here, we focus on the influence of free electrons and neglect higher-order Kerr coefficients, which is called ‘the standard model for nonlinear refractive index saturation' in the case of gases^25^. In the model we assume population is transferred from the valence band to the conduction band through the electric field of the laser pulses^16^. A large fraction of the conduction band population does not remain in the conduction band after local peaks of the laser pulse, but is transferred back to the valence band as the electric field strength decreases. This is sometimes referred to as virtual excitation^19^. The contribution of the current that arises due to the transient conduction band population is included with a two-band model following the work by Földi *et al.*^16^, see Methods section. Critical parameters are the dipole matrix element *d* between the valence and the conduction band and the interband dephasing time *T*_2_. It must be stressed that the underlying model is developed for crystalline solids, while the experiment is carried out with an amorphous sample. For the reasoning, it is referred to the study on optical-field-induced currents in dielectrics^5^ (see especially the Supplementary Material). There it was found that the experimental observations in the strong-field regime did not depend significantly on the crystallinity of SiO_2_. The experimental observations were reasoned on Wannier–Stark localization, which confines the electronic wave function to within a unit cell size, allowing it to explore only the short-scale structure of the lattice^5^.

## Discussion

The inclusion of a transient conduction band population improves the agreement of data and calculation in three points. (i) For pump–probe delays between 0 and 10 fs, the inclusion of a transient conduction band population shifts the probe retardation to later times, closer to the measured probe retardation (Fig. 3a). (ii) The centre frequency of the oscillations shifts from 0.357 PHz (for the calculation without inclusion of a transient conduction band population and for *T*_2_=∞) to 0.360 PHz (for *T*_2_=2 fs). Interpolation of the data points yields a centre frequency of 0.358 PHz (Fig. 3b). (iii) A comparison to a time reference is given by the phase difference between the intensity oscillations due to interference of pump and probe pulses and the oscillations of the probe retardation. Figure 3c shows that the inclusion of a transient conduction band population with a dephasing time of 3.3 fs yields a very good agreement with the measurement.

**Figure 4:**
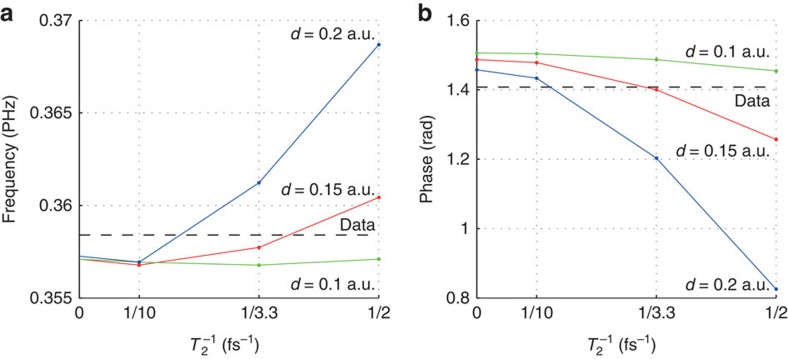
Parameter dependence. The pump–probe delay scan of the probe retardation is calculated for various values for the dephasing time *T*_2_ and for various values for the matrix element *d* (green for *d*=0.1 a.u., red for *d*=0.15 a.u., blue for *d*=0.2 a.u.). The black dashed line marks the experimentally measured value. (**a**) The maximum of the absolute of the Fourier transform (the peak of the curve plotted in Fig. 3b), (**b**) the phase difference to the absolute time reference at a frequency of 0.3556 PHz (the value of the curve plotted in Fig. 3c at a frequency of 0.3556 PHz). The best agreement with the data is obtained for *d*=0.15 a.u. and *T*_2_=3.3 fs.

Through its influence on three observables of the probe retardation as described above, the interband dephasing time *T*_2_ can in principle be determined by comparison of data and calculation. Like many methods of attosecond science, this approach is footed on model assumptions and calculations. A critical point is that the method depends on the choice of the material parameters like the dipole matrix element *d*, see Fig. 4. However, no set of parameters is found that reproduces the measurement perfectly, especially regarding the value of the probe retardation for positive pump–probe delays in Fig. 3a. A reason could be that the mechanisms of delayed Kerr response, transient conduction band population and higher-order Kerr effects would need to be included simultaneously. It is not straightforward to determine the correct value for *d*, because the two-band model employed in this work does not reproduce the high nonlinearity in the ionization yield that is expected^27^,^28^, so the best choice for *d* depends on the laser intensity. Strong-field methods give the correct trend for the ionization yield, but usually do not cover that most of the population transferred from the valence to the conduction band returns back to the valence band at local minima of the electric field strength^14^. *Ab initio* methods are computationally demanding and do not give direct insight in observables that are based on a model assumption, like the dephasing time. Progress in theoretical methods for the description of transient strong-field effects will help to fully exploit the potential of subcycle-resolved probe-retardation measurements.

In conclusion, it is demonstrated that the retardation of a probe pulse through a strong pump pulse in a bulk dielectric can be measured with subcycle resolution in the pump–probe delay. The subcycle dependence reflects the fast dynamics of the nonlinear material response in the strong field. With the assumption that the observed effects do not depend on the crystallinity of the sample (as it was found for the strong-field experiments presented in ref. 5), calculations show that the electronic nonlinearity is revealed both through the Kerr response and through transient conduction band populations. By comparison with model calculations, the method has the potential to determine the interband dephasing time, but progress in the theoretical description of transient strong-field effects is required for reliable estimates. In comparison to other methods in attosecond science, this method poses moderate requirements for instrumentation and hence could make attosecond research accessible for more researchers.

## Methods

### Experimental analysis

**Figure 5:**
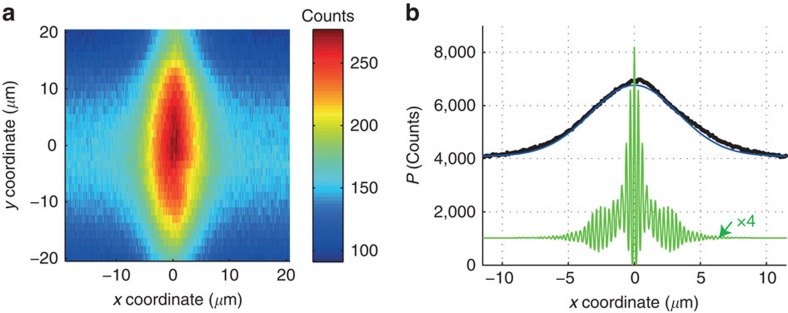
Analysis of the microscope image. The fluorescence from a head-on collision of the probe pulse with a reference pulse inside a cuvette filled with Fluorescein is imaged with a microscope (Fig. 2). Pump and probe pulses propagate along the *x* coordinate. A representative image, recorded at a pump–probe delay *τ*=20 fs, is shown in **a**. The projection *P*(*x*) of the image data onto the *x* coordinate is shown by the black curve in **b**. The base value *P*_base_ is the value of *P*(*x*) outside the region of overlap, for the depicted example *P*_base_≈4,000. The green curve depicts the function *P*_num_(*x*) given by equation (2). The blue curve (which almost perfectly overlaps the black curve) depicts a convolution of *P*_num_(*x*) with a Gaussian function with a s.d. of 3 μm. The blue curve is normalized to the peak value of the black experimental curve, and the *P*_num_(*x*) is scaled by a factor 4 with respect to the blue curve.

The probe retardation *R* is determined from the projection *P*(*x*) of the microscope image onto the *x* coordinate (Fig. 5) using the equation:


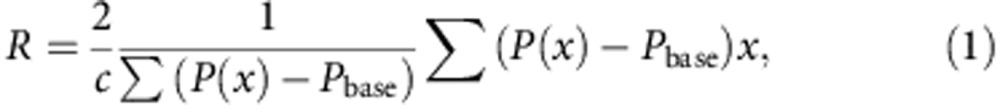


where *c* is the speed of light and *P*_base_ is the base value of the fluorescence (Fig. 5). For infinitely good resolution, the experimental curve *P*(*x*) is expected to follow the function


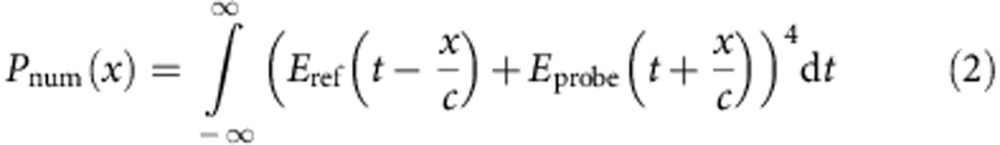


with the waveforms *E*_pump_(*t*) and *E*_probe_(*t*) that are used for the numerical calculations. As shown in Fig. 5, the effective one-dimensional resolution along the *x* direction is about 3 μm, which includes the influence of fluorescence from outside the focal plane. The s.d. of the probe retardation *σ*_*R*_ is affected by Poisson noise in the number of photons that reach the camera, as well as by thermal noise and read noise of the camera. On top of that, mechanical instabilities of the camera mount and the optical mounts have an influence. In principle, *σ*_*R*_ can be made arbitrarily small by using arbitrarily long camera integration times. For the present data, integration times of 50 ms are used. In the absence of precise information about all the influences on *σ*_*R*_, (especially the mechanical instabilities are difficult to estimate), the s.d. is estimated from the experimental values of *R* outside the temporal overlap region (specifically, for pump–probe delays *τ* with −70 fs<*τ*<−50 fs). This yields an estimate of *σ*_*R*_=0.13 fs.

Similarly, the s.d. of the intensity *I*, which is shown in Fig. 2b, could in principle be determined from the camera performance and the photon statistics. However, there are also (presumably dominant) contributions from the shot-to-shot instabilities of the laser. Therefore, the s.d. of the intensity *I* is also estimated from experimental values of *R* outside the temporal overlap region, yielding σ_*I*_=0.8 with respect to the intensity scale used in Fig. 2b.

The experimental uncertainties in the time domain data, characterized by *σ*_*R*_ and *σ*_*I*_, cause uncertainties in the frequency domain analysis shown in Fig. 3b,c. To calculate the error bars shown in Fig. 3b,c, a Monte-Carlo algorithm is used where the Fourier transformation is performed 500 times, each time with Gaussian noise with s.d. *σ*_*R*_ and *σ*_*I*_ added to *R* and *I* before the Fourier transformation is performed.

The interband dephasing time *T*_2_ can in principle be determined by comparison of data and calculation, for example from the phase of the probe retardation depicted in Fig. 3c. For a precise estimate including error bars, the calculation would need to be run with an ensemble of values for the parameter *T*_2_. Since the calculation as described in the next section is computationally very demanding, the calculation was performed only with an ensemble of four values (2, 3.3, 10 fs, ∞) for the parameter *T*_2_. As can be seen from Fig. 3c, the value *T*_2_=3.3 fs yields the best agreement with the data among the ensemble. However, as the agreement with the data is not perfect, Fig. 3c suggests that a slightly higher value would yield an improved agreement. The value *T*_2_=2 fs produces a curve that is outside the experimental error bars, meaning that the lower confidence bound for *T*_2_ is somewhere between 2 and 3.3 fs. The value *T*_2_=∞ produces a curve that is very close to the upper limit of the experimental error bars, meaning that no finite upper confidence bound for *T*_2_ can be stated. However, this method for the estimation of *T*_2_ depends on the choice of the material parameters like the dipole matrix element *d*, see main text and Fig. 4.

### Numerical calculation

The probe retardation is determined by the nonlinear response of the sample to the combined electric field *E*(*t*) of the pump pulse (with field *E*_pump_(*t*)) and the probe pulse (with field *E*_probe_(*t*)) at a finite crossing angle. In traditional nonlinear optics, the nonlinear polarization response *P*_NL_(*t*) to a short pulse is typically described by the electronic and the nuclear (that is, Raman) contribution to the third-order nonlinearity *χ*^(3)^ (for borosilicate glass, we assume a nonlinear refractive index of 1.3 × 10^−13^ e.s.u. (ref. 29)). The electronic response is usually assumed to be instantaneous, and it was shown by femtosecond two-beam coupling that the timescale of the electronic response was well below 1 fs (ref. 23). To test the influence of a potential delay of the electronic nonlinearity, a response time δ*t* is introduced in equation (3). The retarded nuclear response is described by the Raman response function^30^. For the present calculation, the third-order nonlinear polarization is calculated as given in ref. 30:


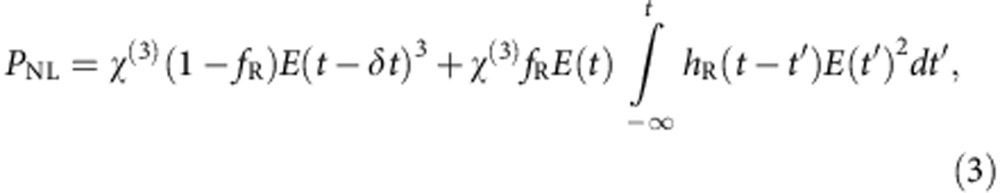


(atomic units are used throughout the paper) where the Raman response function *h*_R_ is given by


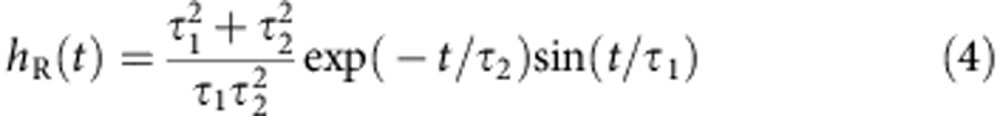


with the parameters *f*_R_=0.18, *τ*_1_=12.2 fs and *τ*_2_=32 fs (ref. 30). The exact values for the Raman response function influence the probe retardation only marginally. To investigate the delay of the Raman response, usually the amplitude change (‘two-beam coupling') is used^21^.

When the electric field of the laser pulse is strong enough to induce a transient conduction band population, the acceleration of the conduction band electrons has to be taken into account. This is calculated using a semiclassical model that was developed by Földi *et al.*^16^. The density matrix in the two-band approximation is given by


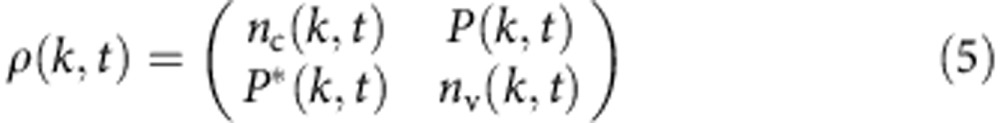


where *n*_c_(*k,t*) and *n*_v_(*k,t*) are the conduction band and the valence band populations, *P*(*k,t*) is the interband coherence. Only the dimension along the laser polarization is considered for the crystal momentum *k*. The time evolution is calculated as


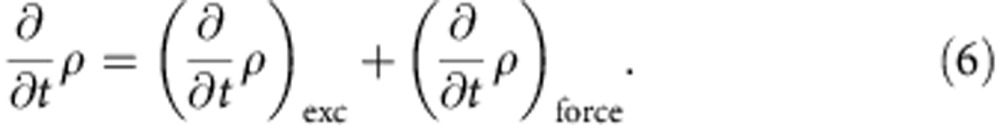


The interband excitation is given by


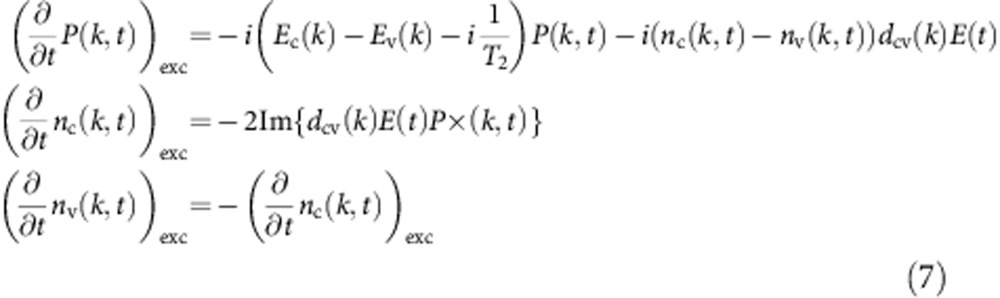


where *E*_v_(*k*) and *E*_c_(*k*) are the energies of the valence and conduction bands:


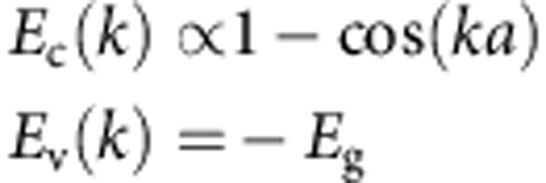


with the band gap *E*_g_=9 eV and the lattice period *a*=0.5 nm. The dipole matrix element *d*_cv_(*k*) is estimated as


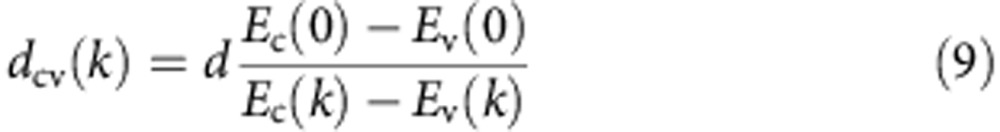


where the values 0.1, 0.15 and 0.2 au are considered for *d* in the calculation (see figure captions), Földi *et al.*^16^ use the value *d*=0.1 au. For the interband dephasing time *T*_*2*_, which describes the decay of the interband coherence, the values ∞, 10, 3.3 and 2 fs are considered in the calculation (see figure captions). Population decay is neglected because it occurs on a timescale above 100 fs (ref. 27).

The acceleration of electrons in the conduction band is described by


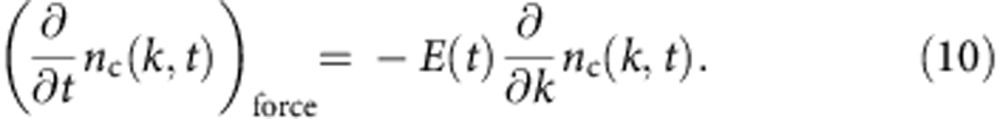


Scattering terms are neglected because they are not correctly reproduced in a one-dimensional model^16^. However, the effect of scattering is likely to influence the probe retardation, especially when the edges of the first Brillouin zone are reached.

The current initiated by the conduction band population is calculated from integration over the first Brillouin zone


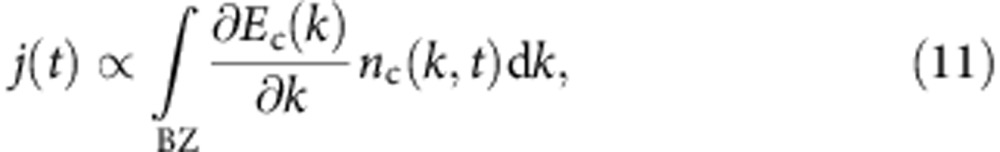


the current in the valence band is neglected. It should be noted that the model used allows also the calculation of the interband polarization^16^, but without capturing the high nonlinearity. Therefore in the present work the interband polarization is included in a phenomenological way through equation (3), which leads to better agreement of data and calculation.

The propagation in the bulk sample along the direction *z* is calculated using the equation


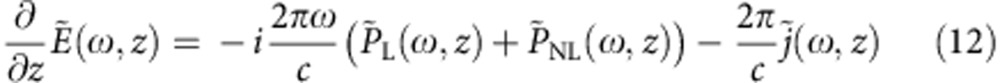


where the convention for Fourier transformation is


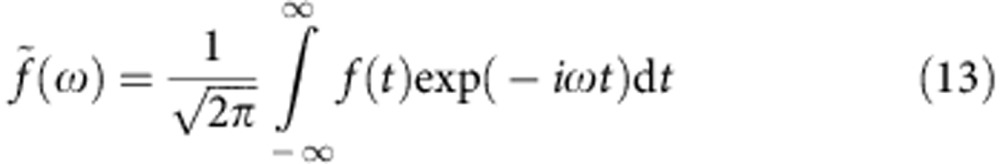


and the linear polarization 
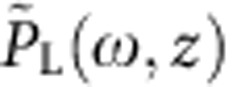
 is calculated using numerical refractive index data.

The beam path from the sample to the cuvette is accounted for by linear pulse propagation for the probe and the reference pulse, and the probe retardation *R* is determined in analogy to the experiment by equation (1). For the parameters used, the calculations show that *R* is in almost perfect agreement with the temporal mean of the probe pulse intensity


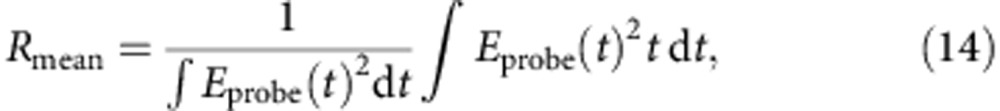


validating the interpretation of *R* as probe retardation.

## Additional information

**How to cite this article:** Pati, A. P. *et al.* Subcycle-resolved probe retardation in strong-field pumped dielectrics. *Nat. Commun.* 6:7746 doi: 10.1038/ncomms8746 (2015).
